# An unusual case of extensive contiguous cervicothoracic spinal tuberculosis involving fourteen damaged segments: A case report

**DOI:** 10.1016/j.ijscr.2020.02.003

**Published:** 2020-02-06

**Authors:** Ifran Saleh, Didik Librianto, Phedy Phedy, Toto Suryo Efar, Anissa Feby Canintika

**Affiliations:** a Department of Orthopaedics & Traumatology, Cipto Mangunkusumo National Central Hospital, Jalan Diponegoro No. 71, Jakarta Pusat, 10430, Indonesia; b Department of Orthopaedics & Traumatology, Fatmawati General Hospital, Jalan RS Fatmawati No. 1, Cilandak Kota Jakarta Selatan, 12430, Indonesia; c Department of Orthopaedics & Traumatology, Faculty of Medicine Universitas Indonesia, Jalan Salemba No. 4, Jakarta Pusat, 10430, Indonesia

**Keywords:** Cervicothoracic spinal tuberculosis, Multilevel contagious involvement

## Abstract

**Introduction:**

Cervicothoracic spinal tuberculosis (CTSTB) is a rare and disabling disease involving the mobile, transitional zone between the lordotic cervical and the kyphotic thoracic spine. Approximately half of those cases involves one or two segments of cervicothoracic vertebrae. We reported a 28-year-old female with tuberculous involvement of fourteen contiguous vertebral segments.

**Presentation of case:**

A 28-year-old female presented with tuberculous involvement of fourteen contiguous vertebral segments is presented. A series of radiographic and CT scan depicted multiple vertebral body destruction anteriorly, along with facet joint dislocation and mild retrolisthesis of C4-C5 segments. MR images of the cervical region was demonstrated pathologic contrast enhancement on C4 to T7 vertebrae, a total of fourteen contiguous segments.

**Discussion:**

Of all spinal tuberculosis, CTSTB accounts for only 5%. In addition to its rarity as a site for tuberculosis, the cervicothoracic junction has anatomical and clinical peculiarities, as a reversal of the mobile-lordotic cervical vertebrae to rigid-kyphotic thoracic vertebrae occurs at this location. Most CTSTB involves only two segments; however, in this case, we found a very extensive case wherein there were fourteen damaged segments.

**Conclusions:**

Our report demonstrates one of the longest involvement of extensive contiguous CTSTB who was treated with one-stage posterior-only approach. However, as this is only a report of one case, further studies are required to investigate the safety and efficacy of such approach for treating extensive CTSTB.

## Highlights

• Cervicothoracic spinal tuberculosis (CTSTB) is a rare and disabling disease involving the cervicothoracic junction.
• Approximately half of these cases involves one or two segments of cervicothoracic vertebrae.
• We reported a 28-year-old female with tuberculous involvement of fourteen contiguous vertebral segments.

## 1 Introduction

Cervicothoracic spinal tuberculosis (CTSTB), defined by tuberculosis infection involving the vertebrae between C7 to T3, is a rare disease that typically results in disabling complications such as kyphotic deformity, large paravertebral abscesses, and progressive spinal cord damage with severe neurological deficit [[1], [2], [3]]. This disease constitutes only 5% of all cases of spinal tuberculosis [4], and it often presents as a treatment challenge for spine surgeons. The cervicothoracic junction is anatomically located in the transitional zone between the more mobile, lordotic cervical and the more rigid, kyphotic thoracic spine [1]. Since the junction is weight-bearing structure, destruction of such structure by tuberculosis infection not uncommonly results in numerous complications aforementioned above. Moreover, the unique interrelationship of cervicothoracic vertebrae commands difficult exposure of the infection focus, both anteriorly and posteriorly, making operative treatment a considerable challenge [1]. Approximately half of cervical spinal tuberculosis involves at most two segments. In this report, we present a case of 28-year-old female diagnosed with CTSTB with involvement of contiguous fourteen vertebrae.

## 2 SCARE criteria compliance

This work has been reported in line with the SCARE criteria [5].

## 3 Case report

A 28-year-old female presented to Fatmawati Hospital, Jakarta, Indonesia due to progressive tetraparesis accounting for seven months. Initially there was mild neck pain, and then she experienced numbness along with weakness of all four extremities. The symptoms were gradually worsened such that at the time of admission, she could not walk.

**Fig. 1:**
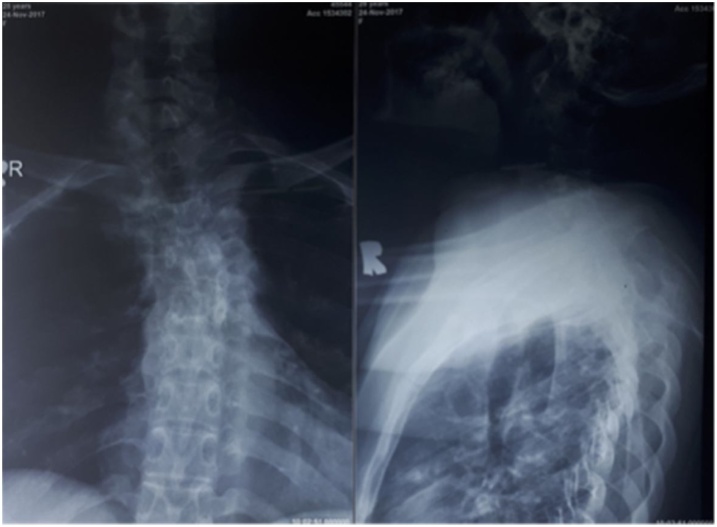
Preoperative radiograph of the patient.

**Fig. 2:**
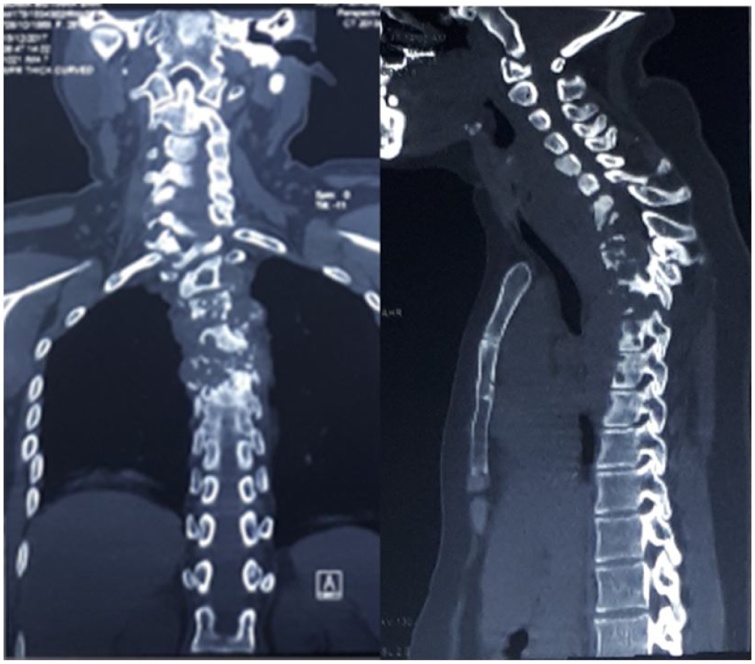
Preoperative CT images demonstrated extensive vertebral body destruction, several facet joint dislocations and mild retrolisthesis of C4 to C5 segment.

**Fig. 3:**
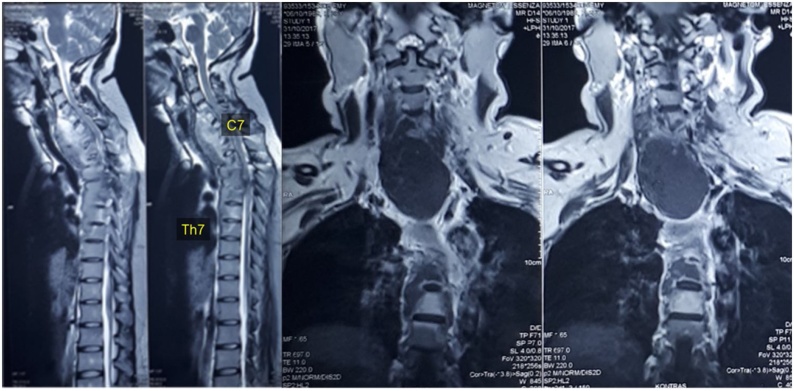
Sagittal cervicothoracic MR images demonstrated pathologic contrast enhancement on C4 to T7 segments along with vertebral body involvement and spinal canal compression at the level of C4 to T4. From coronal MR images, a large paravertebral abscess was presented anteriorly.

A series of radiographic and CT scan depicted multiple vertebral body destruction anteriorly, along with facet joint dislocation and mild retrolisthesis of C4-C5 segments (Figs. 1, 2). MR images of the cervical region was demonstrated pathologic contrast enhancement on C4 to T7 vertebrae, a total of fourteen contiguous segments, with spinal canal stenosis on the level of C4 to T4 and bilateral anterolateral paravertebral soft tissue abscess at the level of C4 to T9 (Fig. 3). Those findings were highly typical of spinal tuberculosis.

**Fig. 4:**
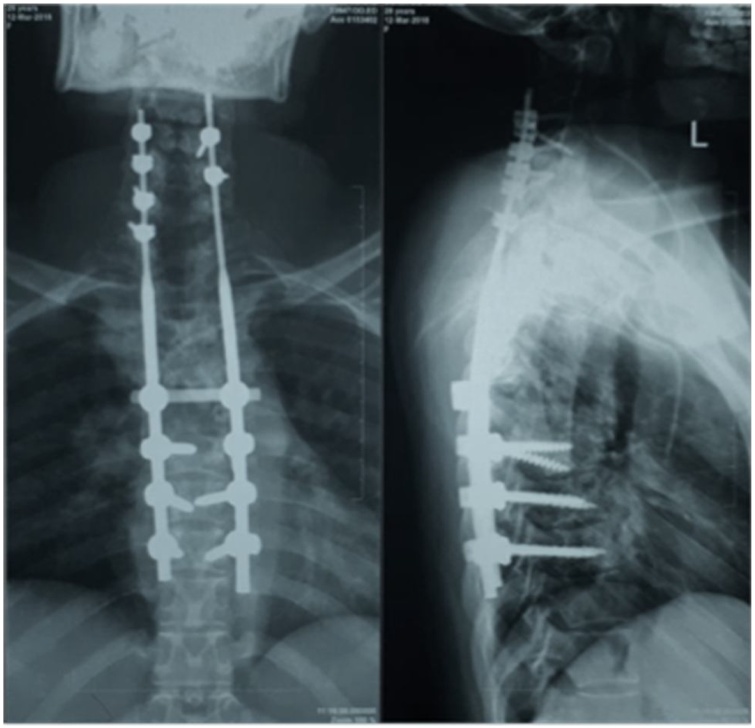
Postoperative radiograph demonstrated pedicle rod and screw construct that resulted in improved kyphotic deformity.

The patient was administered anti-tuberculous agents for two months before undergoing one-staged posterior-only debridement, decompression, and instrumentation. Postoperative radiographs demonstrated that the kyphosis was obviously improved (Fig. 4). After the surgery, the patient was put on a cervicothoracic brace. The patient was closely followed up, and after 12 months postoperatively, she regained neurological recovery with only mild residual neck pain. The patient demonstrated a satisfying level of functional improvement as recorded by the Neck Disability Index (NDI) and SF-36 scores of 4/100 and 94%, respectively.

Bony bridge on CT images was discovered at 18 months of follow-up, along with the normal value of erythrocyte sedimentation rate and C-reactive protein; it was the time we discontinued the anti-tuberculous therapy.

## 4 Discussion

Tuberculosis, caused by *Mycobacterium tuberculosis*, remains one of the oldest diseases worldwide. Despite an ancient disease, tuberculosis remains an important problem, particularly in underdeveloped countries. Extra-pulmonary tuberculosis accounts for 15–20% of all tuberculosis cases [6], and the most common form of this spectrum is spinal tuberculosis, which constitutes of 50% of all skeletal tuberculosis cases [7]. Spinal tuberculosis remains a great challenge to physicians do its nonspecific symptoms that may result in delay of diagnosis, as well as significant high morbidity and mortality.

Of all spinal tuberculosis, CTSTB accounts for only 5% [4]. In addition to its rarity as a site for tuberculosis, the cervicothoracic junction has anatomical and clinical peculiarities, as a reversal of the mobile-lordotic cervical vertebrae to rigid-kyphotic thoracic vertebrae occurs at this location [[8], [9], [10]]. In fact, of all regions of the spine, the cervicothoracic junction is arguably the most challenging entity [11]. Moreover, such junction presumes specific biomechanics and stability different from other spinal regions [9,12]. Affecting mainly to the anterior column, contiguous tuberculous lesion of cervicothoracic junction leads to profound instability and altered biomechanics of the weight-bearing area. CTSTB was also associated with high degree of spinal cord compression that invariably leads to neurological deficit [8].

Most CTSTB involves only two segments; however, in this case, we found a very extensive case wherein there were fourteen damaged segments. In a series of 20 patients with CTSTB, Lan et al. [13] found that 11 (55%) of the patients had two damaged segments, and the most extensive case only involved three segments, which only occurred in one (5%) subject. In a case series of 10 patients, 6 (60%) subjects had three damaged segments, and the most extensive case involved 8 segments. To date, there is no published report regarding CTSTB involving more than ten segments; this is the first report that reports CTSTB involving fourteen damaged segments.

At present, CTSTB is still rarely reported; thus, there has been no specific concensus, let alone for the extensive one. Indications for surgery in CTSTB include kyphosis of ≥ 20°, instability, neurological compromise, and persistent pain [14]. CTSTB could be managed with anterior-only debridement with or without instrumentation, and combined anterior debridement followed by posterior instrumentation. In this case report, we performed one-stage posterior transpedicular debridement, decompression, and instrumentation. Several authors recommended the anterior debridement with or without instrumentation for the main treatment of CTSTB due to its access to tuberculous lesion and its paravertebral abscess, which occurs anteriorly [1,15,16]. However, it is often considered difficult due to the complex structure of the cervicothoracic junction. Many structures, including thoracic bones, clavicles, costal bone, large blood vessels, etc, cover the region [1,9]; thus, the operative field is narrow, which presents a significant challenge to the spine surgeon [17]. Recently, the posterior-only surgery has garnered interests as an alternative treatment for CTSTB, as it is simple, labelled with good clinical efficacy and few complications [9,18]. Feyza et al. [19] and Rath et al. [20] reported good neurological results after posterior debridement and internal fixation in those with neurological impairment due to CTSTB. The results were similar to those obtained via anterior approach. Zeng et al. [9] found that posterior instrumentation may provide better kyphotic correction and is beneficial to the stress dispersion, which can effectively prevent implant failure. Several published studies regarding the management of CTSTB are presented in Table 1.

**Table 1:** Several published studies regarding the management of cervicothoracic tuberculosis.

Author(s)	Year	Country	Study Design	Number of subjects	Diagnosis	Male:Female	Mean Age	Follow Up	Treatment	Outcome
Wu et al. [21]	2019	China	Retrospective	74	Cervicothoracic TB	37:37	24 (range, 5–62 years)	39 (36–96) months	A total of 33 patients underwent one-stage anterior surgery (group A); 16 underwent a combined anterior and posterior surgery (group B) and 25 underwent one-stage posterior surgery (group C).	All groups achieved bone fusion, with pain relief and neurological surgery. Thee surgical strategies significantly improved kyphosis (p < 0.001)
Zhu et al. [4]	2018	China	Prospective	45	Cervicothoracic TB	29:16	35.4 (17–62)	6.6 years on average (range 3–13 years)	19 patients were treated with a single-stage anterior debridement, fusion and instrumentation approach, and the other 26 patients were treated with a single-stage anterior debridement and fusion, posterior fusion and instrumentation approach	The kyphosis angle and NDI and JOA scores were significantly changed from preoperative values of 34.7 ± 6.8°, 39.6 ± 4.6 and 10.7 ± 2.8 to postoperative values of 10.2 ± 2.4°, 11.4 ± 3.6 and 17.6 ± 2.4, respectively (p < 0.05). Aside from one recurrent patient, bone fusion was achieved in the other 44 patients within 6–9 months (mean 7.2 months)
Mahadewa [22]	2016	Indonesia	Case Report	1	Cervicothoracic TB	1:0	12	6 months	One-stage laminmectomy decompression and stabilization fusion via posterior approach	Complete resolution of all neurological deficits except for very mild gait idisturbance
Zhang et al. [1]	2015	China	Prospective	15	Cervicothoracic TB with kyphosis	7:8	40.9 (17–67) years	27.7 ± 8.8	one-stage surgical treatment by posterior fixation, anterior debridement, bone grafting, and anterior fixation	Bone fusion was achieved within three to six months (average, 5.5 months). In the 15 cases, no postoperative severe complications occurred and neurologic function was improved in various degrees.
Lan et al. [13]	2011	China	Prospective	20	Cervicothoracic TB	17:3	N/A	16–39 months	Debridement and bone grafting with internal fixation via anterior approach	Union
Zhang et al. [18]	2011	China	Prospective	10	Cervicothoracic TB with kyphosis	6:4	5.4 ± 1.77	36 (range, 26–47 months)	One-stage posterior focus debridement, bone graft fusion, and instrumentation.	Spinal tuberculosis was completely cured in all ten patients. There was no recurrent tuberculous infection
Ramani et al. [23]	2005	India	Retrospective	61	Spinal TB affecting C3 to D2		Median: 32 (7–68)	38 (24–84) months	Patients with involvement of the C3-C6 vertebrae underwent excision of the involved vertebrae and intervertebral discs followed by reconstruction with titanium implants by anterior approach. A transclavicular approach was used for patients with involvement of the C7-D2 vertebrae.	The neck pain score based on a visual analog scale changed from a preoperative average of 7 to 2 at follow-up after 4 months. 52 (85%) patient had complete relief of pain while 16 patients who had grade III to IV muscle strength regained complete power

In addition to posterior procedures, strut graft or mesh cage could be inserted anteriorly to provide additional stability, especially in our patient whose spinal biomechanics was heavily disrupted from destruction of multiple vertebral segments anteriorly. However, as the risk of graft or cage failure increased along with the increasing number of involved segments, we decided not to perform any procedure anteriorly. To provide additional stability, we put the patient on external brace for six months, afterwards the brace was removed gradually until the involved segments regained bony fusion.

## 5 Conclusions

Our report demonstrates one of the longest involvement of extensive contiguous CTSTB who was treated with one-stage posterior-only approach. However, as this is only a report of one case, further studies are required to investigate the safety and efficacy of such approach for treating extensive CTSTB.

## Sources of funding

None declared.

## Ethical approval

Ethical approval has been received from Fatmawati Hospital, Jakarta, Indonesia.

## Consent

Written informed consent was obtained from the patient for publication of this case report and accompanying images. A copy of the written consent is available for review by the Editor-in-Chief of this journal on request.

## Author contribution

Ifran Saleh: performing the procedure, study concept, providing revisions to scientific content of the manuscript.

Didik Librianto: performing the procedure, data collection, providing stylistic/grammatical revisions to the manuscript.

Phedy Phedy: performing the procedure, data analysis, writing the paper.

Toto Suryo Efar: data collection, writing the paper.

Anissa Feby Canintika: writing the paper.

## Registration of research studies

This study has been registered at researchregistry.com (UIN: researchregistry5169).

## Guarantor

Ifran Saleh.

## Provenance and peer review

Not commissioned, externally peer-reviewed.

## Declaration of Competing Interest

None.
